# Complex Segregation Analysis Provides Evidence for Autosomal Dominant Transmission in the Chinese Han Families with Ankylosing Spondylitis

**DOI:** 10.1155/2017/4515701

**Published:** 2017-12-03

**Authors:** Yutong Jiang, Qing Lv, Shaoqi Rao, Zetao Liao, Pingping Zhang, Mingcan Yang, Qiuxia Li, Shuangyan Cao, Zhiming Lin, Jieruo Gu

**Affiliations:** ^1^Department of Rheumatology, The Third Affiliated Hospital of Sun Yat-sen University, Guangzhou, China; ^2^Department of Medical Statistics and Epidemiology, School of Public Health, Guangdong Medical University, Dongguan, China

## Abstract

**Introduction:**

Familial aggregation of ankylosing spondylitis (AS) has been frequently noticed. However, the mode of inheritance in AS remains poorly understood. Our aim was to determine the mode of inheritance best fitting the observed transmission pattern of AS families.

**Methods:**

Families with 5 or more AS patients diagnosed with 1984 modified New York criteria were recruited. We performed complex segregation analysis for a binary trait in regressive multivariate logistic models. The inheritance models, including sporadic, major gene, environmental, general, and other 9 models, were compared by likelihood ratio tests and Akaike's Information Criterion.

**Results:**

This research included 9 Chinese Han AS families with a total number of 315 persons, including 74 patients. First, familial association was determined. Sporadic with familial association model was rejected when compared with either the general model or the homogeneous general model (*p* < 0.001). The environmental model was also rejected when compared with general models (*p* < 0.02). Mendelian dominate mode fitted best in 5 AS families, while Tau AB free model best explained the mode of inheritance in these AS families.

**Conclusion:**

This study provided evidence in support of Mendelian dominant mode and firstly discovered a non-Mendelian mode called tau AB free inheritance mode in AS.

## 1. Introduction

Ankylosing spondylitis (AS) is a common inflammatory rheumatic disease that mainly affects the axial skeleton and could involve the peripheral joints, entheses, and extra-articular structures, causing characteristic inflammatory back pain, which can lead to structural and functional impairments and a decrease in quality of life [1]. In China, the prevalence of AS has been reported to be 0.11%–0.41% [2].

The etiology of AS remains unclear. Disease susceptibility has been testified to be clearly attributable to genetic factors. From twin recurrence risk studies, the heritability of susceptibility to AS has been estimated at 97% [3]. HLA–B27, encoded in the class I major histocompatibility complex (MHC) region, confers the greatest known susceptibility to AS. The concordance rate for B27-positive dizygotic twins (23%) is much lower than that of monozygotic twins (63%), indicating a large genetic component not linked to the MHC [3]. Besides, there is strong evidence that B27 is not the only MHC gene involved in susceptibility to AS, and other genes such as HLA-B60 are likely to have certain effects on disease susceptibility [4]. Meanwhile, whole genome studies provide strong evidence as to the loci encoding the non-MHC genetic susceptibility to AS [5].

The mode of inheritance of AS has been tested in only several studies. The most convincing suggestion is that AS is oligogenic model with predominantly multiplicative interaction between loci in a sibling recurrent risk studies [6]. Our group confirmed linkage to the HLA-B region in one AS pedigree and reported a new locus in two AS pedigrees located on chromosome 2q36 for the first time with autosomal dominant transmission [7]. Thomson supposed that AS may be closer to addictive or dominant model. Another study on the recurrence of spondyloarthropathy (SpA) among first degree relatives of the patients concluded that no dominance variance and no sex influence were observed in the mode of inheritance in SpA [8]. There is still controversy over the mode of inheritance of AS.

Segregation analysis is often a starting point for family-based genetic studies of complex human diseases (population-based family studies in genetic epidemiology). It could help determine the possible mode of inheritance by comparing relevant hypothesis-based mathematical models on study participants. The objective of this study was to discover the mode of inheritance of AS pedigrees in southern China.

## 2. Methods

Study participants were recruited from Department of Rheumatology in the Third Affiliated Hospital of Sun Yat-sen University. Each patient was assessed by at least two qualified rheumatologists, and the diagnosis was made according to 1984 Modified New York Classification Criteria for AS [9]. Through detailed family history, AS patients with 5 or more patients in his/her family who firstly went to our clinic were included as a possible proband and each family was assumed to be singly ascertained via the sole proband in each family. Inclusion criteria for a family were stated as follows: at least 5 AS patients in the pedigree; at least 3 generations with blood relatives alive; and at least 15 genealogical and nongenealogical relatives in the pedigree. The probands brought the affected relatives to our clinic for further examinations. And three rheumatologists and two nurses drove to the places where a possible proband's family lived. Baseline assessments were completed by trained investigators using identical questionnaires including demographic information (age, gender), disease related characteristics (back pain, morning stiffness, peripheral arthritis, uveitis, etc.), and physical examinations (chest expansion, etc.). Sacroiliac X-ray was performed in suspected members in local hospitals and peripheral blood samples were obtained. All the family members available were recorded for family ascertainment. The concept of uncertainty included at least one of the following criteria: being less than 16 years old with affected blood relatives and having no symptom of AS. Individuals with no reported history, no AS-related clinical manifestations, or no radiographic changes were considered unaffected. Totally 9 families with a total number of 315 individuals were recruited. There were 74 AS patients in total, including 43 male patients and 31 female patients, together with 4 males and 3 females with uncertain disease status. This study was conducted in compliance with the Helsinki Declaration to protect human subjects and was approved by the Third Affiliated Hospital of Sun Yat-sen University ethics committee. All the participants gave written informed consent.

### 2.1. Statistical Analyses

Complex segregation analysis was performed by using the program SEGREG within the SAGE 6.3 program package. The segregation model for a binary trait assumes that susceptibility to disease, *γ*, defined as the probability that an individual is affected with disease, depends on an unobserved latent factor termed type, designated as *u*, which can take on one of the three values AA, AB, or BB [10]. Type is best defined in terms of the expected distribution of an individual's offspring and genotypes are the special case of types. The incorporation of types introduces two sets of parameters, type frequencies and transmission parameters [11]. If the type frequencies are in Hardy-Weinberg equilibrium proportions, then they are defined in terms of frequency of component A. Transmission parameters, recorded as *τ*_AA_, *τ*_AB_, and *τ*_BB_, refer to the probabilities that individuals of types AA, AB, and BB transmit the component A (allele, if the type is a genotype) to offspring, respectively. The corresponding baseline parameters for susceptibility are *β*_AA_, *β*_AB_, and *β*_BB_.

For a binary trait, the susceptibility *γ* is given by the cumulative logistic function

(1) $$\begin{array}{c} \gamma =\frac{e^{\theta \left(i\right)ti}}{1+e^{\theta \left(i\right)}}, \end{array}$$

where *ti*, the analysis trait of the *i*th individual, is 1 for an affected individual and 0 for an unaffected individual; and *q*(*i*), the logit of the susceptibility for the *i*th individual, can depend on both major type (*u*) and covariate *xi*1; *xi*2; …; *xi ***p**:

(2) $$\begin{array}{c} \theta u\left(\;i\;\right)=\beta u+\xi 1xi1+\cdots +\xi pxip. \end{array}$$

The segregation models can be classified according to the number of baseline parameters for susceptibility in the model: one susceptibility type (no segregation, *β*_AA_ = *β*_AB_ = *β*_BB_), two susceptibility types (dominant or recessive Mendelian transmission, *β*_AA_ = *β*_AB_, or *β*_AB_ = *β*_BB_), and three susceptibility types (*β*_AA_, *β*_AB_, *β*_BB_) need to be estimated individually.

#### 2.1.1. One Susceptibility Type Models

One susceptibility type includes sporadic model without or with familial association in the absence of transmission of a major gene. Familial association involving father-mother (FM), mother-offspring (MO), father-offspring (FO), and sib-sib (SS) is recorded as *δ*_FM_, *δ*_MO_, *δ*_FO_, and *δ*_SS_. Because there were insufficient data to estimate the father-mother association (it was frequently not possible to maximize the likelihood), we set it equal to 0 for all these models [10]. In sporadic model without familial association, all these familial association and transmission of major gene are not assumed, meaning that *δ*_FO_ = *δ*_MO_ = *δ*_SS_ = 0. There is only one baseline parameter *β*_AA_  ( = *β*_AB_ = *β*_BB_) which is interpreted as the natural logarithm of the odds of susceptibility versus nonsusceptibility to the disease in the absence of other factors [12]. According to different familial association, sporadic model with familial association is subdivided into three subtypes. The first model has the same familial association (*δ*_FO_ = *δ*_MO_ = *δ*_SS_). The second model has the same FO and MO association, while three familial associations need to be estimated in the third one, respectively.

#### 2.1.2. Two Susceptibility Type Models

Two susceptibility type models include Mendelian dominant model and Mendelian recessive mode, both meeting Mendelian transmission in which *τ*_AA_ = 1, *τ*_AB_ = 0.5, and *τ*_BB_ = 0. In Mendelian dominant model, baseline susceptibility parameters for AA and AB are constrained equal, while *β*_AB_ was set equally to *β*_BB_ in Mendelian recessive model.

#### 2.1.3. Three Susceptibility Type Models

All the baseline susceptibility parameters for AA, AB, and BB need to be estimated in three susceptibility models, involving several transmission models. Above all, general model allows the transmission probabilities *τ*_AA_, *τ*_AB_, and *τ*_BB_ to take on any arbitrary values between 0 and 1. As all other models are nested in general model, we can use general model as the baseline model to compare with other models with individual parameters. A more restricted general transmission model (homogenous general model) assumes homogeneity of the phenotypic distribution across generations and must satisfy two conditions: the type frequencies must follow Hardy-Weinberg equilibrium proportions and *τ*_AB_ must be equal to a specific function of the frequency of A, *τ*_AA_, and *τ*_BB_; thus, only two of the transmission probabilities can be freely estimated [11]. Environmental model assumes that the segregation of a certain disease is purely due to environment factors, instead of major gene effect. Then there is no transmission from parents to off-spring, indicating that three types transmit equally to the allele A frequency (*τ*_AA_ = *τ*_AB_ = *τ*_BB_ = *q*_A_). Tau AB free model assumes that the transmission parameter for AB can be freely estimated within the range of 0-1, while *τ*_AA_ and *τ*_BB_ are constrained to 1 and 0, respectively. This model does not meet Mendelian transmission rules. The following models are all Mendelian models with other different parameters. Major gene without familial association model refers to the transmission of a major gene without any familial association. Mendelian codominant model with familial association assumes the transmission of a major gene and familial association [12]. Mendelian additive model has the baseline susceptibility parameter for AB constrained to the mean score of *β*_AA_ and *β*_BB_. Mendelian decreasing model assumes the decreasing tendency of the baseline susceptibility parameter for AA, AB, and BB as *β*_AA_ ≥ *β*_AB_ ≥ *β*_BB_. Similarly, Mendelian increasing model has increasing baseline susceptibility parameter for AA, AB, and BB as *β*_AA_ ≤ *β*_AB_ ≤ *β*_BB_.

Hypotheses are assessed by the likelihood ratio test, under the assumption that the negative of twice the difference in natural logarithms for hierarchical models follows a *χ*^2^ distribution [13]. The degree of freedom (d.f.) is equal to the difference of the number of parameters estimated in both models. The rejection of a model tested compared to the general model means the hypothesized model does not fit the data.

The most parsimonious model is identified by using Akaike's Information Criteria (AIC) [14], which is defined as [−2ln⁡(*L*) + 2(number  of  parameters  estimated)]. The model with a smaller AIC refers to better fitting in the data.

## 3. Results

### 3.1. Characteristics of Pedigrees

A total of 9 pedigrees were included in the study and the average size was 35.00 (SD, 8.67). Of all the 315 individuals, 169 were male and 146 were female patients, while affection status for 4 males and 3 females was unknown. Individuals with unknown status were retained in the data to establish relationship within pedigrees but were not used in the analysis because their phenotype values were set to missing. The number of generations of each pedigree varied from 3 to 5 generations. Detailed distribution of relationship types was described in Table 1. The ratio of the percentage of males affected (25.4%) to that of female affected (21.2%) was about 1.20.

First, to determine support for familial association, we compared sporadic model without familial association together with sporadic models with three types of familial association.  Table 2 presents the effect of incorporating different familial association in these nonsegregating models. Sporadic models with any familial associations (Models 2, 3, and 4) significantly fitted better than sporadic model without familial association (Model 1), with *p* value less than 0.001. Next, among Models 2, 3, and 4, Model 2 fitted best with a less AIC value and fewer degrees of freedom indicating few parameters estimated. Therefore, it provided support for the existence of familial association in the data and three equal familial/multifactorial components significantly improved the fitness. So we set familial association parameters equally in the subsequent models.

Table 3 presents the estimated parameters for different models of inheritance fit to the entire sample. Sporadic with familial association model was rejected when compared with either the general model or the homogeneous general model (*p* < 0.001) and the other sporadic models were also rejected (not shown in Table 3), indicating the existence of a major gene. Besides, the purely random environmental model was also rejected when compared with either the general model (*p* = 0.012) or the homogeneous general model (*p* = 0.005), meaning that there was transmission of a major gene in the pedigrees. However, models with Mendelian transmission were all rejected (*p* < 0.001). The homogenous general model was not rejected (*p* = 0.60). Among nongeneral models, only tau AB free model was not rejected compared to the general model, with *p* value of 0.24. These results presented that tau AB free model was the best-fitting model. The same methods were used for analyzing the mode of inheritance of these AS families individually and the results showed that 5 of the 9 pedigrees fitted best in Mendelian dominant mode (not shown in the table).

## 4. Discussion

The mode of inheritance in familial AS was assessed by complex segregation analysis in this study. Because AS has a relatively low prevalence, there have been very few studies with large pedigrees concerning the mode of inheritance. Here, we included 9 AS families with a total number of 74 patients and rechecked disease status of each individual. And we proved again that Mendelian dominant mode fitted the mode of transmission in Chinese Han AS families [7].

We first confirmed familial associations contributing to the susceptibility of AS, as three equal familial components significantly improved fitness by comparing sporadic models. Then, no major effect was testified by significant rejection of the sporadic model compared with the general model. Meanwhile, the environment model (no transmission model) was also rejected compared with the general model. Both hypotheses of “no major gene effect” and “no transmission of major gene effect” were rejected in support of segregation of major gene(s) [15, 16].

AS is among complex human diseases. In complex phenotypes, the effect sizes of the involved alleles are likely to vary and could range from minimal to significant. Many alleles are expected to exert modest effects which may not be apparent based on current phenotyping and genetic approaches [17]. On the other hand, multiple variants with large effect sizes could be the main determinants of heritability of the complex diseases. When a single allele exerts a great effect on the phenotype, familial segregation will follow a Mendelian pattern of inheritance. However, contribution of additional variants to the phenotype can give variable expressivity or incomplete penetrance [18]. Commonly, familial aggregation of a complex trait usually does not follow a clear pattern of segregation.

In our study, Mendelian inheritance pattern was rejected, while tau AB free model fitted the best. The obvious difference between two models was whether transmission parameter for AB was set to 0.5. Sibling recurrent risk studies suggested that AS could be an oligogenic model with predominantly multiplicative interaction between loci [6]. The study also pointed out that the sibling recurrence risk ratio was not significantly greater than the parent-child recurrence risk ratio, suggesting that there was not a great dominance variance component to susceptibility to AS. That meant substantial non-MHC genetic or other MHC susceptibility genes except HLA-B27 may take part in the susceptibility to AS. And another study also found that multiplicative interaction existed between HLA and non-HLA genetic components [8]. Another reason could be that the trait does not segregate in accordance with Mendel's rules. The proportions of phenotypes observed do not match the predicted values in several conditions, including gene conversion [19], genomic imprinting [20], trinucleotide repeat expansion [21], cytoplasmic inheritance [22], and infectious heredity [23]. In addition, a large number of alleles are expected to cosegregate with the phenotype, which may cause deviation from Mendelian transmission pattern.

Even in the absence of Mendelian inheritance, family-based genetic analysis in families offers a robust approach for identification of the causative variants for complex phenotypes [17]. What we can do next is to gather more AS families, to focus on family members that are more distantly related but are phenotypically affected. The approach by reducing the number of shared alleles is expected to enhance identification of causal variants. Another practical approach is to sequence and contrast sequence data from family members comparing patients with different disease activity [24] or clinical manifestations. Further investigation involving linkage analysis, direct DNA sequencing, and Genome-Wide Association Studies (GWAS) could also be conducted to identify the causative alleles in families [25].

## 5. Conclusions

This study provided evidence in support of Mendelian dominant mode for a second time and firstly discovered a non-Mendelian mode called tau AB free inheritance mode in AS Chinese Han pedigrees, laying a foundation for further genetic studies of familial AS aggregation.

## Figures and Tables

**Table 1:** Distribution of relationship types in the study sample.

	All	Patients/concord affected pairs
Total	315	74
Male	169	43
Female	146	31
Parent-offspring	443	65
Sib-sib	317	73
Sister-sister	101	12
Brother-brother	74	32
Sister-brother	142	32
Grandparental	340	13
Avuncular	840	38
Cousin	811	54

**Table 2:** Parameter estimated in sporadic model, incorporating various familial associations by multivariate logistic model (*δ*_FM_ = 0) in nine AS families.

Parameters	Model 1	Model 2	Model 3	Model 4
	*δ*_FO_ = *δ*_MO_ = *δ*_SS_ = 0	*δ*_FO_ = *δ*_MO_ = *δ*_SS_	*δ*_FO_ = *δ*_MO_, *δ*_SS_	*δ*_FO_, *δ*_MO_, *δ*_SS_
*β*	−1.15	−1.58	−1.71	−1.69
*δ*_FO_	[0]^§^	2.64	2.73	2.58
*δ*_MO_	[0]	2.64	2.73	2.76
*δ*_SS_	[0]	2.64	3.38	3.24
−2ln⁡(*L*)	339.65	284.88	284.24	284.12
d.f.^△^	1	2	3	4
*p* ^*∗*^	—	<0.001	<0.001	<0.001
AIC	341.65	288.88	290.24	292.12

^§^Parameters were fixed at the values indicated; ^△^d.f., degree of freedom; ^*∗*^compared with model 1.

**Table 3:** Parameter estimated from segregation analysis in various models in nine AS families.

Parameter	Model 2	Model 5	Model 6	Model 7	Model 8	Model 9	Model 10	Model 11	Model 12	Model 13
	Sporadic	General	Homogenous	Environment	Tau AB free	Codominant	Dominant	Recessive	Additive	Decreasing
*β*_AA_	−1.58	−36.28	−36.28	−3.85 × 10^9^	−3.01	−0.80	−0.80	−168.85	−0.80	−0.80
*β*_AB_	−1.58	−1.20	−1.20	−1.53	−0.89	−0.80	−0.80	0.27	−77.75	−0.80
*β*_BB_	−1.58	−1.95 × 10^5^	−4295.02	−1.53	−6846.23	−154.61	−154.70	0.27	−154.70	−154.70
*q*_A_	2.64	0.19	0.19	0.0002	0.18	0.17	0.17	0.93	0.61	0.17
*τ*_AA_	—	0.9997	0.998	0.0002	[1]^§^	[1]	[1]	[1]	[1]	[1]
*τ*_AB_	—	0.00	0.00	0.0002	0.999	[0.5]	[0.5]	[0.5]	[0.5]	[0.5]
*τ*_BB_	—	0.9995	0.99	0.0002	[0]	[0]	[0]	[0]	[0]	[0]
−2ln⁡(*L*)	284.88	193.50	193.78	204.44	196.39	253.86	253.88	253.97	263.04	253.89
d.f.	2	8	7	5	6	5	4	4	4	5
*p* ^*∗*^	<0.001	—	0.60	0.012	0.24	<0.001	<0.001	<0.001	<0.001	<0.001
AIC	288.88	203.50	207.78	214.44	206.39	263.86	261.88	261.97	271.04	263.89

^*∗*^Compared with Model 5 (general model); ^§^parameters were fixed at the values indicated; Model 2 refers to sporadic model with equal family association parameters; Mendelian increasing model cannot be estimated with the parameters settled in the model.
